# Liposome-mediated macrophage reprogramming: emerging strategies for chronic disease therapy

**DOI:** 10.3389/fimmu.2025.1653642

**Published:** 2025-10-17

**Authors:** Hongnv Zhang, Nan Hu, Zhenghao Wang, Shaodong Zhai

**Affiliations:** ^1^ Third Hospital of Shanxi Medical University, Shanxi Bethune Hospital, Shanxi Academy of Medical Sciences, Tongji Shanxi Hospital, Taiyuan, China; ^2^ Institute of Medical Technology Research, Shanxi Medical University, Taiyuan, China

**Keywords:** liposome, macrophage, chronic diseases, nanomedicines, inflammation

## Abstract

With the rapid advancement of nanotechnology, the application of nanomedicine has become increasingly widespread, demonstrating remarkable potential for highly precise targeting and efficacious drug delivery. Compared to conventional drug delivery approaches, nanomedicine effectively addresses issues such as nonspecific drug distribution and severe adverse effects, significantly enhancing therapeutic efficacy through its targeted delivery mechanisms. As an innovative drug delivery vehicle, liposomes exhibit tremendous application potential owing to their outstanding biocompatibility, extensive applicability, remarkable ability to improve drug stability and bioavailability, precise targeting capabilities, membrane structures that facilitate drug permeation, and high degree of tunability. In the field of chronic disease management, liposomes serve as sophisticated vehicles for targeted and controlled drug delivery, offering innovative therapeutic approaches for various chronic conditions. Macrophages, which play a pivotal role in modulating inflammatory responses and promoting tissue repair, have emerged as crucial targets for alleviating inflammatory symptoms. Nevertheless, achieving precise and efficient targeting of macrophages remains a significant challenge in current research. This article systematically reviews recent advances in liposome-based therapies for chronic diseases, including cardiovascular diseases, cancers, chronic respiratory diseases (e.g., chronic obstructive pulmonary disease, pulmonary fibrosis, and asthma), and metabolic disorders (e.g., diabetes), with particular emphasis on the therapeutic potential of liposomes in modulating macrophage activity. Furthermore, it summarizes and analyzes the major challenges and obstacles currently faced in liposome research, providing novel insights for future research directions and facilitating the translation of research findings into clinical applications.

## 1 Introduction

Chronic diseases (non-communicable diseases, NCDs) have long courses and complex causes, involving the interaction of genetic, environmental and behavioral factors. These diseases mainly include cardiovascular diseases, malignant tumors, chronic respiratory diseases (e.g., pulmonary fibrosis, chronic obstructive pulmonary disease (COPD), and asthma), and metabolic disorders (e.g., diabetes). Accounting for approximately 80% of all NCD-related deaths, they represent the leading cause of global disability and mortality (1, 2). The World Health Organization (WHO) predicts that by 2030, the mortality rate of NCDs will account for 70% of the total global deaths (3). Moreover, the cumulative economic losses from 2011 to 2030 are expected to reach 47 trillion US dollars (the above four categories account for 64%) (4). The pathogenesis of these diseases is closely related to macrophage dysfunction: abnormally activated macrophages in cardiovascular diseases drive atherosclerosis (5); tumor-associated macrophages in malignant tumors promote cancer progression and metastasis (6); macrophages mediate chronic inflammation and fibrosis in respiratory diseases (7); and they are directly involved in insulin resistance in metabolic disorders occur (8). Therefore, targeting the regulatory mechanism of macrophages will enable the development of innovative therapeutic interventions and significantly alleviate the global health burden and socio-economic pressure.

In the occurrence and development of chronic diseases, macrophages, serving as the core effector cells of the innate immune system and the “immune sentries” of the body’s homeostasis, play a crucial triple regulatory role in inflammatory responses, pathogen clearance, and tissue repair owing to their unique polarization plasticity (9–12). They recognize pathogen-associated molecular patterns (PAMPs) and damage-associated molecular patterns (DAMPs) through pattern recognition receptors (PRRs), thereby initiating a precise dual regulatory program: (1) pro-inflammatory M1-type macrophages secrete TNF-α, IL-6 and CCL2, and generate nitric oxide (NO) via inducible nitric oxide synthase (iNOS) to enhance pathogen clearance ability; (2) reparative M2-type macrophages highly express arginase-1 (Arg-1), cluster of differentiation 36 (CD36), and nuclear factor erythroid 2-related factor 2 (Nrf2), secrete anti-inflammatory factors such as IL-10 and TGF-β, and promote tissue remodeling and fibrosis (13–17). This dynamic balance in M1/M2 polarization states constitutes the core mechanism through which macrophages coordinate inflammation control, pathogen clearance and tissue repair.

However, in chronic pathological environments (e.g., tumor microenvironment), this balance is frequently disrupted. Notably, DAMPs exhibit complex bidirectional effects: they can not only initiate protective immune responses but may also, due to excessive accumulation, exacerbate inflammation, reshape the microenvironment, and accelerate disease progression (18) (19). In tumors, infiltrated tumor-associated macrophages (TAMs) mainly present a pro-tumor M2-like phenotype and drive tumor progression by promoting angiogenesis, metastasis, immunosuppression, and matrix remodeling (20) (21). The plasticity of macrophages (including TAMs) between inflammation and repair, as well as between promoting and resisting disease, and their core regulatory roles make them highly attractive targets for therapeutic interventions through reprogramming their polarization states.

Against this backdrop, the rapid development of nanotechnology has pushed nanomaterials to the forefront of biomedicine. Among them, liposomes, as the most mature and clinically verified nanocarriers, have demonstrated unique advantages in targeted therapy. Since Bangham et al. discovered the phospholipid vesicle structure in 1965 (22), liposomes have developed into biomimetic membrane delivery systems (as evidenced by multiple U.S. Food and Drug Administration (FDA)-approved formulations) (23, 24). The core features include: (1) an amphiphilic phospholipid bilayer structure (25) that enables hydrophobic drug incorporation into lipid membranes while encapsulating hydrophilic drugs within aqueous cores (26), achieving flexible adaptation to dual drug-loading modalities; (2) multiple functional advantages including targeted delivery capability (through surface engineering for lesion-specific targeting) (27), excellent biocompatibility and biodegradability, low toxicity and immunogenicity, as well as prolonged drug circulation time and enhanced stability (28, 29). These characteristics establish liposomes as an ideal platform for regulating the pathological microenvironment, with demonstrated applications in the following areas: cardiovascular diseases—precisely delivering therapeutics to plaque-resident macrophages to attenuate inflammatory progression (27); oncotherapy—targeting TAMs to reprogram M2 polarization and reverse immunosuppression (30); pulmonary diseases—enhancing pulmonary tissue bioavailability through alveolar macrophage-targeted sustained drug release (31); and diabetic ulcers—modulating macrophage polarization to promote wound healing (32).

**Figure 1:**
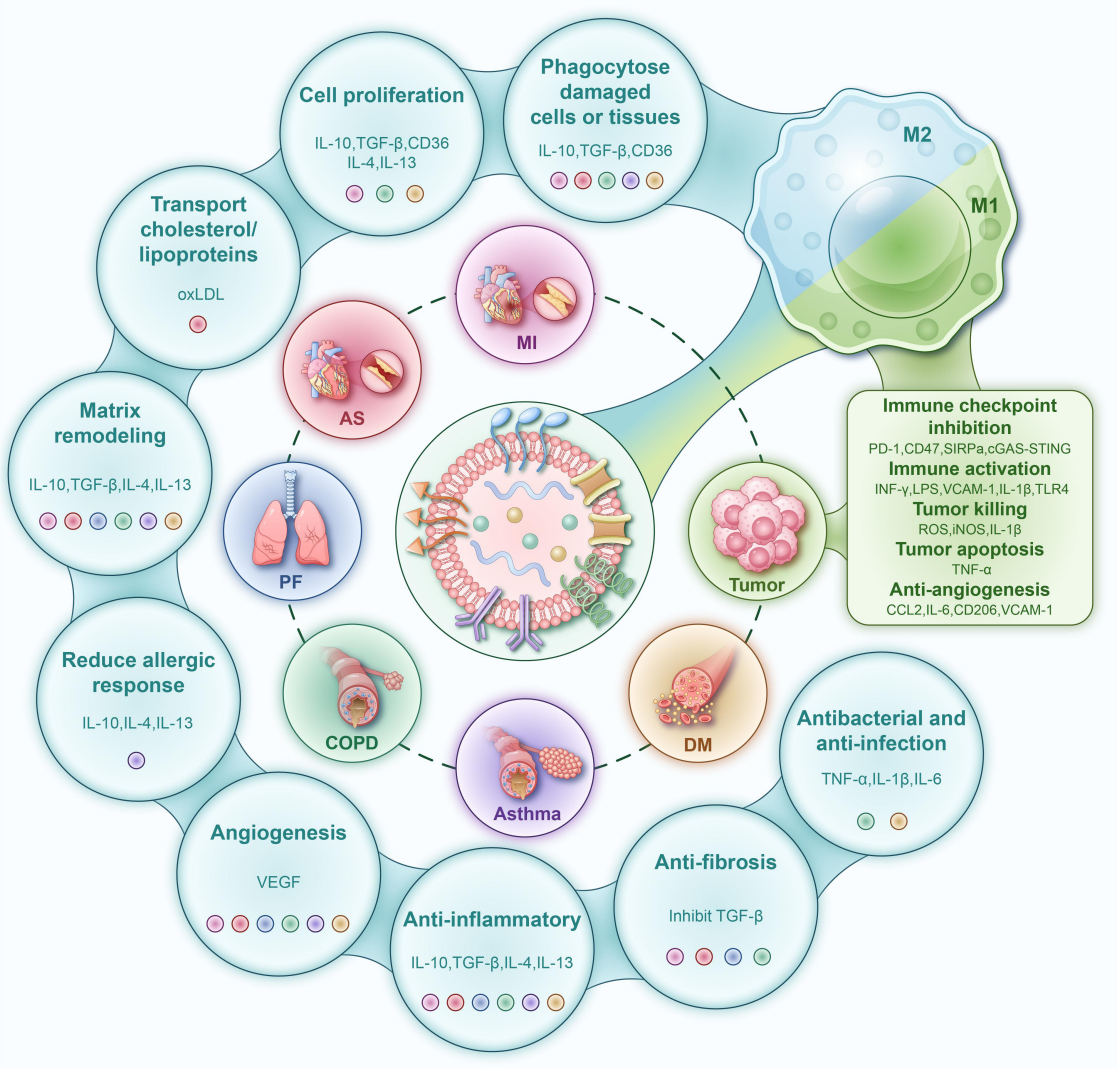
Schematic diagram of the molecular mechanisms by which engineered liposomes modulate macrophage polarization for the treatment of chronic diseases. Promotion of M1 Polarization (Right Panel): Liposomes drive macrophages toward a pro-inflammatory, anti-tumor M1 phenotype. Activated M1 macrophages upregulate the expression of IL-1β, TNF-α, IL-6, iNOS (producing NO), and ROS through pathways such as NF-κB, mediating tissue damage and tumor apoptosis. Concurrently, they secrete CCL2 and IL-6 and upregulate VCAM-1, thereby inhibiting angiogenesis and recruiting additional immune cells. Promotion of M2 Polarization (Left Panel): Liposomes drive macrophages toward an anti-inflammatory and pro-repair M2 phenotype. Activated M2 macrophages exert multiple functions including inflammation suppression (via IL-10, TGF-β, IL-4, IL-13), phagocytosis of damaged cells (mediated by CD36, IL-10, TGF-β), promotion of cell proliferation (via IL-10, TGF-β, CD36, IL-4, IL-13), matrix remodeling (through IL-10, TGF-β, IL-4, IL-13), reduction of allergic responses (by IL-10, IL-4, IL-13), anti-fibrotic effects (via TGF-β inhibition), and antibacterial/anti-infection activities (mediated by TNF-α, IL-1β, IL-6). Additionally, the M2 phenotype facilitates physiological angiogenesis through VEGF-mediated mechanisms. PD-1, Programmed cell death protein 1; CD47, Cluster of Differentiation 47; SIRPα, Signal Regulatory Protein α; cGAS-STING, cyclic GMP-AMP Synthase-Stimulator of Interferon Genes; INF-γ, Interferon-gamma; LPS, Lipopolysaccharide; VCAM-1, Vascular Cell Adhesion Molecule 1; IL-1β, Interleukin-1 beta; TLR4, Toll-Like Receptor 4; ROS, Reactive Oxygen Species; iNOS, inducible Nitric Oxide Synthase; TNF-α, Tumor Necrosis Factor-alpha; CCL2, C-C Motif Chemokine Ligand 2; IL-6, Interleukin-6; CD206, Cluster of Differentiation 206; CD36, Cluster of Differentiation 36; IL-10, Interleukin-10; TGF-β, Transforming Growth Factor-beta; IL-4, Interleukin-4; IL-13, Interleukin-13; oxLDL, oxidized Low-Density Lipoprotein; VEGF, Vascular Endothelial Growth Factor.

Liposome-based nanotherapeutic strategies represent an innovative intervention approach for the treatment of chronic diseases by precisely regulating the balance of macrophage M1/M2 polarization. Engineered liposomes achieve efficient delivery to pathological sites through surface-modified targeting moieties (e.g., carbohydrates, peptides, antibodies, and proteins) and accurately drive macrophage phenotype switching via loaded immunomodulators. In cancer treatment, they promote M1 polarization to enhance anti-tumor immunity; in cardiovascular and respiratory diseases, they induce M2 polarization to accelerate tissue repair; and in diabetes, they modulate the M1/M2 balance to improve insulin sensitivity and facilitate wound healing. Given the crucial role of macrophages in chronic inflammatory diseases, this review systematically analyzes the application mechanisms of liposomes in four major areas: cardiovascular diseases, malignant tumors, chronic respiratory diseases, and metabolic disorders (**Figure 1**). It critically discusses how liposome platforms leverage macrophage biology to achieve targeted therapeutic effects, while also outlining current challenges and future directions in the field, providing new perspectives for advancing the clinical application of liposome-based therapies in the management of chronic diseases. Although this review aims to cover major chronic diseases, the more extensive body of research in oncology is emphasized, reflecting both the historical dominance and ongoing innovation of liposome technology in the field of cancer therapy.

## 2 Cardiovascular diseases

Cardiovascular diseases (CVD) have become a major global health burden (33–35). Although surgical operations are effective for some patients, they carry inherent surgical risks (36–38). To address these clinical challenges, liposome nanocarriers have been developed as a promising therapeutic alternative (22, 39, 40). The core therapeutic mechanism lies in precisely regulating the function of macrophages - which is precisely the key pathogenic link of cardiovascular diseases.

Myocardial infarction (MI) results from ischemic necrosis of the myocardium due to coronary artery occlusion (41). However, reperfusion therapy, as the cornerstone therapeutic strategy, presents a paradoxical dilemma. While restoring blood flow is essential, the reperfusion process itself precipitates a cascade of pathological events, including reactive oxygen species (ROS) burst, intracellular calcium overload, and maladaptive inflammatory responses. These mechanisms collectively contribute to the demise of otherwise salvageable cardiomyocytes (termed ischemia-reperfusion injury, IRI), with sustained inflammatory activation serving as the central pathogenic driver (42, 43). Consequently, effective modulation of inflammatory responses remains a major therapeutic challenge in myocardial infarction and reperfusion injury. Targeting the central pathological feature of macrophage polarization imbalance, research groups have investigated novel liposome-based delivery strategies. For instance, Tan et al. (44) constructed platelet-mimicking liposomes (PLP), which precisely delivered microRNA-21 (miR-21) to circulating monocytes via membrane fusion, driving M2 polarization and improving cardiac function. This strategy is minimally invasive and highly targeted, but it relies on the overlap of monocyte recruitment timing and the window period of the enhanced permeability and retention effect (EPR), which may limit its clinical applicability. Dong et al. (45) developed spleen-targeted liposomes (ST-MT@lipo2) to reduce inflammatory cell migration by regulating the heart-spleen axis monocyte chemoattractant protein-1/C-C chemokine receptor type 2 (MCP-1/CCR2) pathway. However, the size-dependent targeting efficiency of the nanoparticles cause inconsistent therapeutic effects, and the heterogeneity of the spleen microenvironment may pose off-target risks. Similarly, Cheng et al. (46) designed isogenic repair macrophages (PS-c@M) to restore immune homeostasis by synergistically inhibiting the STING pathway and repairing mitochondrial function. Their advantage lies in low immunogenicity and long-term retention characteristics, but the complexity of the preparation process and high production costs make it difficult to meet clinical demands. Additionally, Tan et al. (47) adopted a synergistic strategy of transgenic macrophages combined with CD47 antagonists, which can restore efferocytosis and block the “do not eat me” signal. However, the potential immunogenicity and long-term safety of gene editing have not been fully verified. Weng et al. (48) developed a ROS-responsive RvD1 delivery platform that achieves inflammation-targeted controlled release through a biomimetic platelet membrane. The challenge lies in the need to adapt the ROS response threshold to different pathological gradients, and the biological half-life limitation of RvD1 still needs to be overcome. Despite these strategies breaking through the limitations of insufficient targeting and single-pathway regulation of traditional therapies, they are still mired in three major translational quagmires. The mass production crisis of complex carriers, the safety black hole of gene/biomimetic materials, and the common predicament of dynamic pathological response mismatch.

Given the central role of macrophages in infarct repair and their dual value as therapeutic targets and drug delivery vehicles, Che et al. (49) revealed an innovative mechanism for the uptake of methotrexate liposomes (MTX-liposomes) by target cells, as a process dependent on a precisely regulated neutrophil-mediated cascade transport system. This study found that neutrophils carry MTX-liposomes and undergo physiological changes, safely releasing the nanocarriers into target macrophages through a strictly controlled cell lysis process, thereby achieving precise drug delivery and efficient utilization. This neutrophil-mediated delivery strategy exhibits remarkable adaptability and holds promise for application in myocardial ischemia-reperfusion injury (MIRI) models. Another groundbreaking study (50) demonstrated that biomimetic neutrophil liposomes (Neu-Lipos) not only reduce the number of proliferating macrophages but also significantly lower the levels of key pro-inflammatory cytokines, thereby improving the myocardial repair process. The strategy of inducing macrophage polarization toward a regenerative phenotype has emerged as a highly promising therapeutic approach for ameliorating post-myocardial infarction remodeling. Also, miR-21 plays a pivotal role in regulating macrophage polarization, Tan et al. (44) developed a novel platelet membrane-coated nano-delivery system. This system employs miR-21-loaded mesoporous silica nanoparticles as the core, enveloped by a fusion of platelet membranes and cationic liposomes. The innovative design enables specific targeting of macrophages in cardiac inflammatory sites, releasing miR-21 for anti-inflammatory regulation. It effectively protects cardiac function in mice with myocardial ischemia-reperfusion injury and precisely modulates macrophage polarization states.

Atherosclerosis (AS), a prevalent cardiovascular disorder, is profoundly influenced by hemodynamic factors such as shear stress and vascular bifurcation geometry (27). The pathological process is characterized by a triad of key features, including endothelial dysfunction, chronic inflammation, and lipid-rich plaque formation (51–54). The progressive nature of AS ultimately leads to luminal stenosis or complete occlusion, resulting in compromised blood flow and subsequent ischemic tissue damage in downstream vascular beds. Although the early lesion microenvironment is more amenable to intervention (55, 56), its asymptomatic and insidious nature causes diagnostic difficulties and delays treatment (54, 57–59), urgently requiring early precise diagnostic and therapeutic strategies. Traditional anti-inflammatory therapies are limited by non-specific distribution, poor water solubility, and dose toxicity (e.g., bleeding, kidney damage) (60–63). While nanocarrier delivery systems show the potential to address these limitations and achieve targeted lesion treatment (64, 65).

In the field of macrophage-targeted therapy for atherosclerosis, the lipid-mediated reprogramming strategy through multi-dimensional mechanisms demonstrates breakthrough potential. Dong et al. (66) developed HA-modified hybrid liposomes that reverse M1-to-M2 macrophage polarization and promote lipid metabolism via autophagy activation and CD36 downregulation, thereby enhancing plaque stability. Separately, Zhang et al. (67) designed a similar system for dual-targeting (plaque/macrophage) delivery, demonstrating efficacy in mitigating endothelial dysfunction and reprogramming macrophage phenotype to attenuate atherosclerosis. For microenvironment regulation, researchers have developed a targeted liposome delivery system (68) that innovatively exploits macrophage metabolic pathways to catalyze nitric oxide (NO) production. This system demonstrates dual therapeutic mechanisms by mitigating endothelial cell senescence and scavenging ROS, while simultaneously inhibiting the VEGF signaling pathway to suppress pathological angiogenesis. The integrated approach enables dynamic modulation of the plaque microenvironment. In response to the problem of cholesterol reverse transport, the targeted liposome developed by Shen et al. (69) significantly promotes cholesterol efflux and effectively clears ROS through drug synergy, simultaneously up-regulating the ATP-binding cassette transporter A1/G1 (ABCA1/G1) pathway and inducing macrophage polarization to M2 type, thereby achieving significant plaque clearance effects. While Yang et al. (70) pioneered the macrophage membrane hybrid liposomes, which take a different approach by regulating the BDH1/ORM1/RPS27L to form a metabolic-inflammation-stress response network, inhibiting the ferroptosis process, effectively blocking the positive feedback loop of lipid peroxidation and inflammation. While these technological advances uniformly exhibit key advantages including precise targeting capability, multifunctional therapeutic synergy, and excellent biocompatibility, their clinical translation remains hindered by challenges in complex carrier production processes.

**Figure 2:**
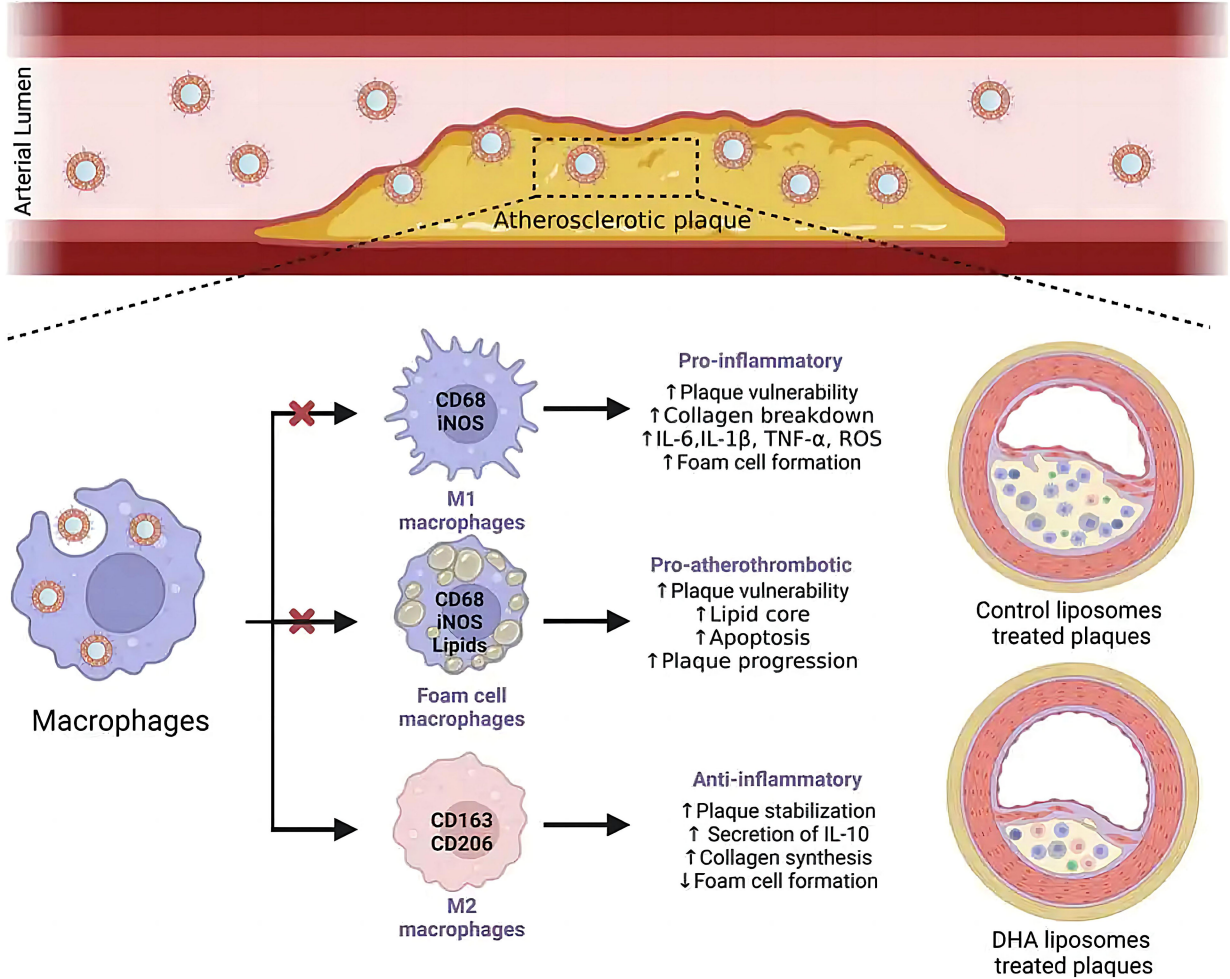
Liposome-encapsulated DHA targeting plaques after intravenous administration, uptake by macrophages, and improvement of atherosclerosis (77).

Docosahexaenoic acid (DHA) demonstrates pleiotropic therapeutic effects against atherosclerosis, particularly through its potent anti-inflammatory, antioxidant, and antiproliferative activities (71–76). Notably, this omega-3 fatty acid exhibits synergistic potential with liposomal delivery systems, which preferentially accumulate in plaque-resident macrophages. Chong et al. demonstrated that DHA-loaded liposomes are efficiently internalized by activated macrophages, triggering robust anti-inflammatory and antioxidant responses while effectively suppressing foam cell formation-a critical step in atherosclerotic plaque progression (**Figure 2**) (77). Mechanistically, intravenously administered DHA-liposomes exhibit selective homing to macrophage-rich atherosclerotic lesions, where they promote phenotypic reprogramming of these immune cells. Preclinical studies suggest that intravenous DHA-liposome delivery represents a pharmacologically superior approach compared to oral administration, offering enhanced bioavailability with minimal adverse effects (78). While these findings position liposomal DHA as a promising therapeutic strategy, further clinical translation is necessary to validate its efficacy and safety in human subjects. This targeted delivery paradigm not only improves drug bioavailability but also reduces systemic exposure, potentially overcoming the limitations of conventional small-molecule therapies. The ability to precisely modulate macrophage polarization through liposomal delivery opens new avenues for immunomodulatory approaches in cardiovascular disease management.

On the other hand, liposomes serve as versatile biomimetic platforms that can be strategically engineered to emulate biological membrane functions, thereby enabling innovative modulation of macrophage behavior (79, 80). Wu et al. developed an innovative apoptotic body-mimetic liposomal system that faithfully replicates the natural targeting properties of apoptotic vesicles. This system demonstrates remarkable precision in delivering anti-inflammatory payloads to atherosclerotic macrophages, achieving triple therapeutic benefits including inflammation modulation, plaque stabilization, and potential application for inflammatory comorbidities (81). Building on membrane-mimetic technology, P-Lipo was created by Song et al. through an extrusion-based fusion of conventional liposomes with platelet membranes (82). This biohybrid system retains native platelet targeting capabilities while gaining enhanced drug delivery functions. *In vitro* studies using RAW264.7-derived foam cells demonstrated that P-Lipo maintains multifunctional adhesion properties and exhibits selective accumulation in atherosclerotic lesions. The platform’s multivalent targeting capacity and biocompatibility enable effective intervention in macrophage-driven atherosclerosis without detectable toxicity, representing a significant advancement in both therapeutic efficacy and safety profiles. Further innovating this approach, Sha et al. developed macrophage membrane-cloaked nanoparticles by enveloping liposomal cores with native macrophage membranes (83). These biomimetic nanotherapeutics operate through a competitive binding mechanism *in vivo*, effectively scavenging pathogenic molecules (ox-LDL and LPS) that would normally be internalized by macrophages. This intervention achieves dual therapeutic effects including substantial reduction in foam cell formation (by up to 68% in murine models) and significant suppression of pro-inflammatory cytokine expression. The most advanced iteration of this technology, MP-QT-NP, demonstrates unprecedented therapeutic potential through a multi-modal mechanism (84). These biomimetic platforms collectively represent a paradigm shift in atherosclerosis treatment, offering targeted therapeutic strategies that address multiple pathological pathways simultaneously. The successful translation of these technologies could revolutionize clinical management of atherosclerotic cardiovascular disease.

## 3 Cancer

Cancer remains one of the most complex and challenging diseases in medical research, presenting ongoing therapeutic difficulties (85, 86). Liposomes have emerged as particularly promising drug delivery systems in cancer therapy due to their unique phospholipid bilayer structure, which provides exceptional drug encapsulation and delivery capabilities (87). These versatile nanocarriers can simultaneously transport multiple therapeutic agents including chemotherapeutic drugs (88–92), antigens (93–96), antibodies (97–99), and immunomodulators (100–106), enabling precise and synergistic therapeutic effects. Furthermore, liposomes demonstrate excellent compatibility with physical treatment modalities such as photothermal, photodynamic, and radiotherapy approaches, significantly enhancing their therapeutic potential. Through physical regulation mechanisms, liposomes allow precise control over their stability and permeability, enabling spatiotemporal regulation of drug release rate and locations. This controlled release ensures optimal drug concentrations in tumor tissues while minimizing leakage into normal tissues, thereby significantly improving drug bioavailability and therapeutic outcomes.

### 3.1 Liposomal co-delivery of immunomodulators for macrophage-based cancer immunotherapy

Liposomes serve as intelligent platforms that integrate chemotherapy and immunotherapy to modulate macrophages, thereby generating synergistic therapeutic benefits. For instance, the TSPLs system enhances lung targeting through the co-delivery of paclitaxel and rSEC2 while activating T-cell subsets to reverse immunosuppression (107). Similarly, a liposomal formulation combining oxaliplatin and STING agonists promotes immunogenic cell death (ICD), thereby enhancing antigen presentation and T-cell infiltration (91). Furthermore, the NPCD@ALN system significantly improves the therapeutic efficacy against osteosarcoma by synergistically inducing pyroptosis and ICD (108). The success of these strategies hinges on the sophisticated integration of the EPR effect with active targeting technologies to improve targeting accuracy. Additionally, spatiotemporally controlled release enables coordinated action between chemotherapeutic agents and immunomodulators, ultimately activating antitumor immunity. It is particularly noteworthy that such designs transcend the limitations of conventional chemotherapy, elevating liposomes from simple drug carriers to multifunctional regulators of the tumor immune microenvironment.

**Figure 3:**
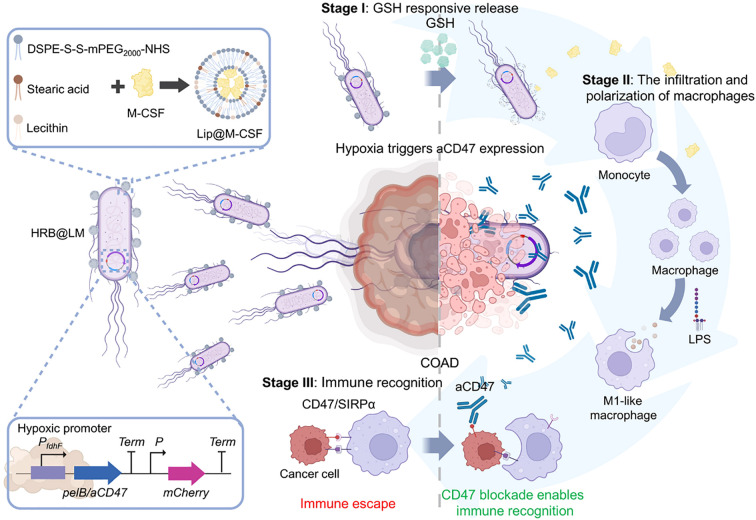
Hypoxia-responsive HRB@LM system targets CD47/SIRPα signaling to synergistically activate macrophage-T cell antitumor immune cascades (112).

Liposome technology has made breakthrough progress in the field of antigen/antibody targeted delivery, demonstrating a powerful ability to precisely regulate the tumor immune microenvironment. In terms of targeting mechanisms, the bionic liposomes (TSPLs) of 4T1 cancer cell membrane hybridization have achieved precise co-delivery of chemotherapy drugs and immunomodulators through homologous targeting (107). Meanwhile, the CAR-T exosome fusion system (Lip-CExo@PTX) innovatively uses bispecific scFv to simultaneously target tumor antigens and immune checkpoints (109). These designs ingeniously leverage the natural targeting characteristics of biological systems, organically integrating the passive targeting EPR effect with the active targeting molecular recognition. In terms of immune regulation, the synergistic use of STING agonist liposomes and CD40 antibodies (110), as well as membrane fusion liposomes (MFL) targeting apoptotic bodies (111), significantly enhanced antigen presentation efficiency through spatiotemporal precise immune stimulation. Particularly worthy of attention are the designs of the nano-liposome-bacterial hybridization system (**Figure 3**) (112) and the protease-responsive eLipo (113). The former utilizes the biosynthetic ability of bacteria to achieve *in situ* expression of antibodies, while the latter overcomes the targeting barrier through microenvironmental response release. In the treatment of immunologically “cold” tumors like microsatellite-stable colorectal cancer (MSS-CRC), engineered cationic liposomes simultaneously enhance RNA m6A methylation through FTO protein inhibition and silence the CD47 immune checkpoint, effectively driving M2-to-M1 TAM repolarization while boosting macrophage phagocytic activity (114). Innovative “tail-flipped” nanoliposomes mimicking peroxidized phospholipids specifically target SR-B1 receptors on M2-TAMs to deliver STAT6 inhibitors, effectively disrupting pre-metastatic niche formation (115). Metabolic intervention strategies further expand the therapeutic scope, exemplified by PEGylated liposomes co-delivering mannose (glycolysis inhibitor) and levamisole (mitochondrial function blocker) to synchronously modulate cancer cell and TAM metabolism when combined with radiotherapy (116). These results not only address the key issues of traditional therapies such as poor targeting and high toxicity, but also achieve the integration of “delivery and activation” through engineering design, providing new ideas for tumor immunotherapy. However, to achieve clinical translation, challenges such as vector stability, large-scale production, and the precision of immune regulation still need to be addressed. The future development directions may focus on the optimization of intelligent response systems, the development of multi-target collaborative delivery strategies, and the establishment of individualized treatment plans, etc.

Liposome technology has made contribution to PD-1/PD-L1 immunotherapy, primarily through the optimization of delivery strategies. ThioLipos developed by Shin et al. demonstrated significant monotherapy effects in colon cancer models by inhibiting FoxM1-mediated PD-L1 expression (117). This finding suggests that targeting the upstream regulatory factors of PD-L1 may be more advantageous than direct blocking. The BLN liposomes (118) demonstrated the significance of microenvironment regulation by inducing calmodulin exposure and macrophage polarization, and the NGR liposomes (105) achieved the dual effects of vascular normalization and PD-L1 down-regulation. The combined use of FAK inhibitors with liposomal doxorubicin (119) and the ozone-liposome enhanced radiotherapy technology (120) both demonstrate that tumor antigens produced by ICD can establish a self-reinforcing anti-tumor immune cycle. It is worth noting that metabolic reprogramming demonstrates unique value, which includes L-arginine metabolism (121), tryptophan metabolism (122), and iron metabolism (123). These studies suggest that future immunotherapy may need to adopt a “multi-pronged” strategy: blocking immune checkpoints, improving the tumor microenvironment (TME), activating innate immunity and reshaping the metabolic microenvironment at the same time. This comprehensive intervention approach might offer a new breakthrough in overcoming the current problem of drug resistance in immunotherapy.

Liposomes have emerged as a key platform for overcoming immunosuppression and enhancing anti-tumor immunity by efficiently regulating the TME through various innovative strategies as carriers of STING agonists. This system not only achieves the synergistic delivery and controlled release of drugs (91), but also directly reshapes the composition and function of the immune microenvironment through ingenious design: optimizing lipid composition to enhance lysosomal escape and type I interferon production (124). Intelligent responsive liposomes (ultrasound (125), pH (126) and enzyme responses (127) can achieve tumor site-specific STING activation, significantly enhancing treatment specificity. Furthermore, through targeted modification and multi-mechanism synergistic strategies, the liposome-STING agonist system has demonstrated a powerful potential for precise regulation of the TME. By integrating synergistic strategies such as photodynamic therapy, ferroptosis induction and STING activation (128) or exosome- liposome hybridization system (129), multiple immunosuppressive links in the TME can be targeted simultaneously, establishing a self-reinforcing anti-tumor immune cycle. Mitochondria-directed liposomes, BQR@MLipo, induce ferroptosis-specific HMGB1 release via DHODH inactivation, accompanied by mtDNA leakage that activates the cGAS-STING pathway, driving CD8^+^ T cell infiltration (130). These advancements highlight the significant value of STING-loaded agonist liposomes in coordinating innate and adaptive immune responses, addressing tumor heterogeneity, and reversing drug resistance. Future research should focus on enhancing the clinical translational ability of these complex systems and exploring their precise application in the regulation of individualized immune microenvironments.

### 3.2 Physically stimulated liposomes for macrophage-based cancer immunotherapy

**Figure 4:**
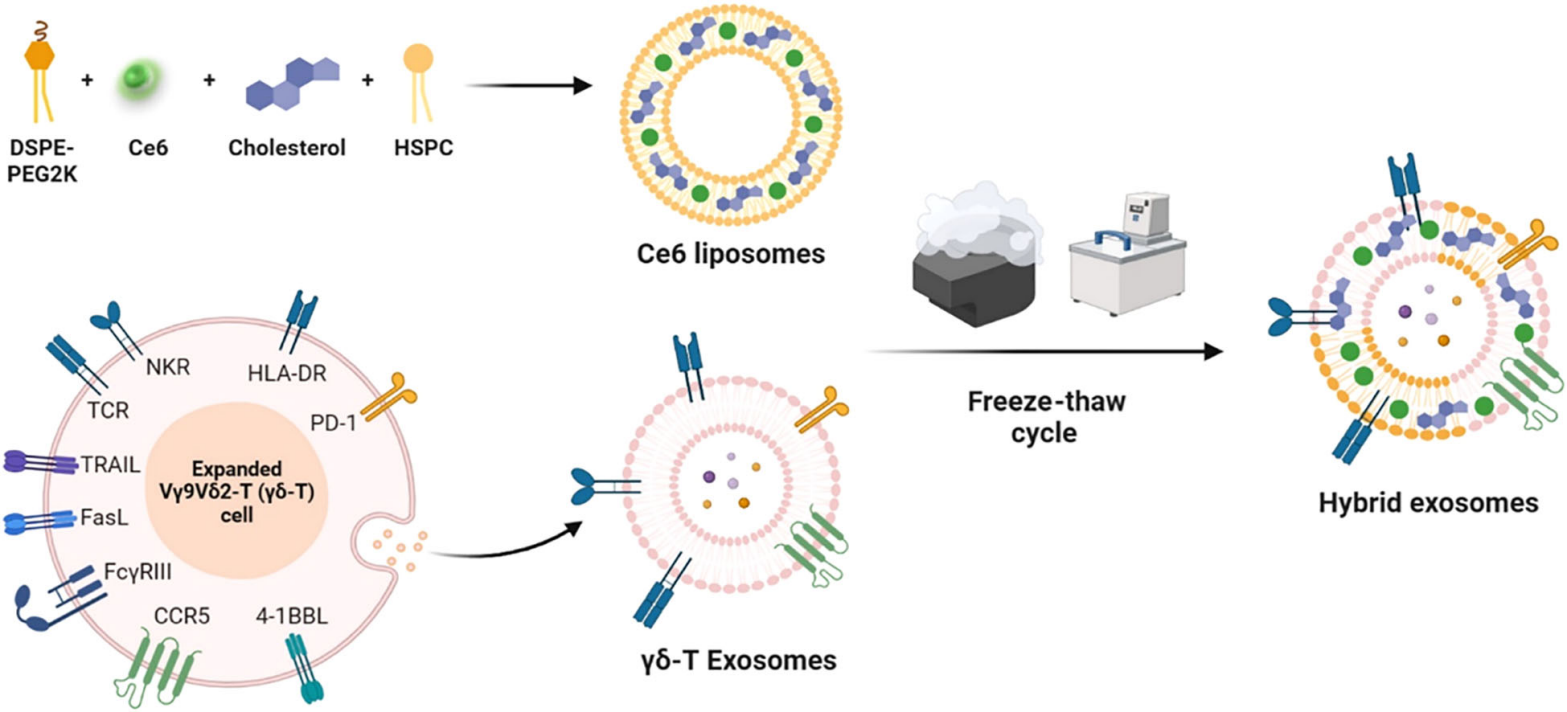
Schematic diagram of photobiological immunotherapy for melanoma based on the fusion of γδ-T exosomes and Ce6 liposomes (133).

Liposomes have significantly advanced the development of combined tumor immunotherapy strategies through the integration of photothermal therapy (PTT) and immune microenvironment regulation. A T-cell membrane-fused liposomes (TMVL-I) and M1 macrophage-bacterial outer membrane hybrid systems (RB@OL), overcome the spatiotemporal limitations of conventional therapies by leveraging biomimetic targeting and photothermal-immunological synergistic mechanisms, enabling precise immune activation against both primary and metastatic tumors (131, 132). On the other hand, liposomes serve as a delivery platform for photodynamic therapy (PDT), significantly expanding the therapeutic dimensions of PDT through precise modulation of ICD and tumor microenvironment remodeling. In melanoma treatment, a γδ-T exosome-modified Ce6-TEXO system enables targeted delivery mediated by CCR5/PD-1. Under 660–700 nm light irradiation, it generates ROS and synergizes with exosomal granzyme/perforin to induce ICD, releasing DAMPs such as CRT/ATP, thereby effectively activating CD8^+^ T cells (**Figure 4**) (133). This strategy lies in the integration of cell membrane-targeting technology with the immune-activating properties of PDT, achieving a spatiotemporal synergistic enhancement between exosomes and PDT. In the development of *in situ* vaccines, an endoplasmic reticulum-targeting liposome (Par-ICG-Lipo) fabricated using microfluidic technology to achieve high drug loading, induces ER-specific ICD under near-infrared light irradiation. Through the release of tumor-associated antigens (TAAs) and DAMPs, this process effectively transforms the tumor into an endogenous vaccine (134). This design overcomes the limitations of traditional vaccine preparation and demonstrates the unique advantages of PDT in initiating *in situ* immunity. To address the drug-resistant microenvironment, the Pt/Ce6-LP (135) depletes GSH through Pt(IV) prodrug conversion, alleviates hypoxia, and modulates ROS levels, thereby driving TAM repolarization towards the M1 phenotype and establishing long-term immune memory. This approach successfully triples the efficacy of PDT, proving that metabolic modulation and remodeling of the immune microenvironment can effectively reverse tumor drug resistance (136).

Meanwhile, the synergistic therapy combining liposome and radiotherapy (RT) is evolving from a traditional physical radiosensitization strategy toward a new paradigm focused on remodeling the immune microenvironment. Central to this shift is leveraging the immunogenic effects of RT, achieved through precisely engineered liposomal delivery systems that enable multi-level modulation of the cancer-immunity cycle. During the immune initiation phase, RT not only directly induces ICD in tumor cells but also acts synergistically with intelligent lipid-based systems. For instance, the Lipo-Ele@CuO_2_ liposome developed by Jiang et al. utilizes RT to trigger cuproptosis, markedly enhancing the release of DAMPs, while simultaneously reprogramming immunosuppressive TAMs, thereby establishing a potent “*in situ* vaccine” effect (137). The Cold Exposure-SL liposome system leverages RT-induced burst generation of peroxynitrite to enhance oxidative stress and suppress myeloid-derived suppressor cells (MDSCs), thereby creating a favorable microenvironment for immune activation (138). At the effector phase of immunity, tailored strategies designed for specific tumor microenvironments have demonstrated distinct advantages. In glioblastoma, an MMP-2-responsive liposome (D@MLL) (139) leverages RT-enhanced blood-brain barrier permeability to synergistically promote M1-type TAM polarization, effectively countering the immunosuppressive microenvironment. Meanwhile, the IR-LND@Lip nano-adjuvant developed by Zhou et al. achieves synergistic activation of the cGAS-STING pathway under radiotherapy, converting immunologically “cold” tumors into “hot” phenotypes, while simultaneously blocking immune checkpoint signals such as PD-L1 and TGF-β (140). Another innovative approach by Suo et al. involved TAFL biomimetic liposomes that exploit exosomal fusion properties to specifically target cancer stem cells (CSCs), releasing aspirin to induce CSC apoptosis and suppress stemness while utilizing photothermal therapy to alleviate hypoxia and indirectly reduce M2-TAM-derived immunosuppressive signals, thereby creating synergistic RT-immune modulation (141).

These advances signify a fundamental transformation in the role of liposome-based platforms in cancer therapy. Originally used merely as radiosensitizers in radiotherapy or as simple carriers for agents in photothermal/photodynamic therapy, they have now evolved into integrated multifunctional systems capable of simultaneously modulating tumor metabolism, the immune microenvironment, and cell death. By incorporating strategies such as spatiotemporal regulation of the STING pathway, these multimodal systems successfully achieve cascaded conversion of physical energy to chemical energy and then to biological effects. This not only enhances local therapeutic ablation but also drives the reprogramming of systemic anti-tumor immunity. This shift marks a strategic transition in cancer treatment paradigms from traditional “single-target inhibition” to a new era of “multimodal intervention”. The core breakthrough lies in the precise temporal control of DAMPs release and immune cell reprogramming, establishing an integrated framework of that progresses from *in situ* immune priming to microenvironment remodeling and finally to a systemic anti-tumor response. Current research is advancing the transition from laser-mediated local treatments to systemic immunomodulatory strategies, offering novel avenues to overcome the challenges in solid tumor therapy. Future efforts should focus on achieving precise matching between individualized drug delivery systems and radiotherapy regimens, as well as leveraging artificial intelligence and other technologies to optimize spatiotemporal treatment parameters, ultimately enabling comprehensive regulation from local irradiation to system-wide immune control.

## 4 Respiratory diseases

Chronic respiratory diseases, accompanied by structural abnormalities of the airways and lungs, pose a major global public health challenge, with continuously rising burdens of morbidity and mortality (142, 143). Among these, pulmonary fibrosis is characterized by persistent activation of myofibroblasts, excessive extracellular matrix deposition, and chronic inflammatory cell infiltration (144–146). COPD is primarily manifested as irreversible airflow limitation (147), and asthma is marked by recurrent episodes and acute exacerbations (148). Approximately 4 million annual deaths are attributed to these diseases, resulting in a substantial societal burden (149). In recent years, liposome-based strategies targeting the regulation of macrophages have achieved a series of advances in chronic respiratory disease treatment research.

**Figure 5:**
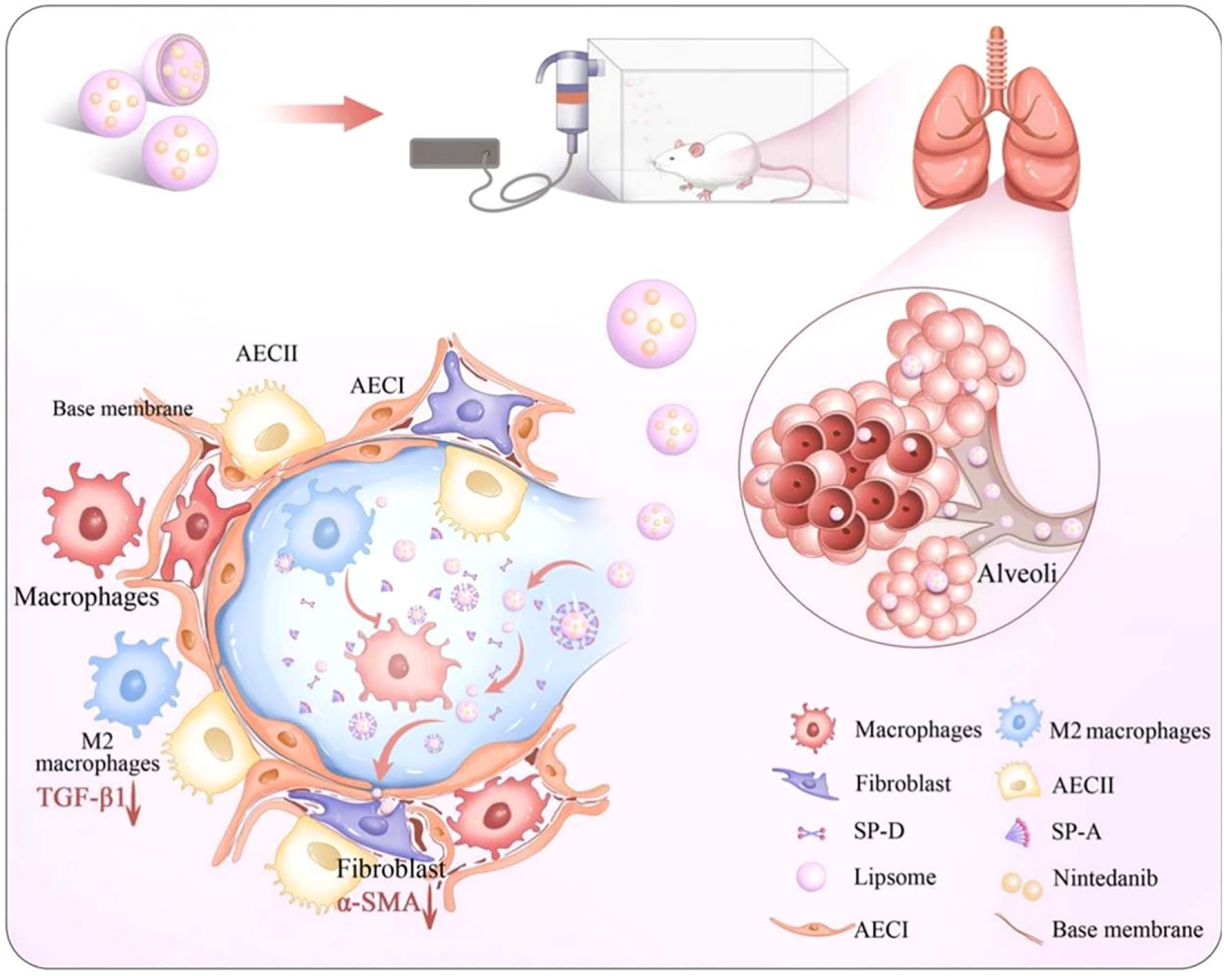
Macrophage involvement in PF therapy. Impact of Nin-lipo on M2 macrophage polarization and lung fibrosis (153).

In the field of pulmonary fibrosis, Peng et al. (150) and Cheng et al. (151) collectively confirm the critical influence of liposomal physicochemical properties on delivery efficiency from the perspective of liposome design. Liposomes constructed with saturated neutral and anionic phospholipids exhibit high stability and pulmonary permeability, When loaded with salvianolic acid B, they achieve therapeutic effects by inhibiting inflammation and imbalances in the coagulation-fibrinolysis system. In contrast, a Gal3 siRNA-loaded liposome that intervenes in the pathological crosstalk among endothelial cells, macrophages, and fibroblasts by blocking the Gal3-TGFBR1/TLR4 signaling axis. This targeting strategy provides a new paradigm for precise regulation of intercellular communication based on optimized liposome physicochemical properties. Notably, macrophage polarization regulation has emerged as a core strategy in multiple research efforts. The NAMPT drives M2 polarization through a non-enzymatic activation of STAT6 signaling, while clodronate liposome-mediated macrophage depletion and reconstitution experiments revealed the central role of monocyte/macrophage populations in fibrosis (152). Nin-lipo is a biomimetic liposome that mechanically interferes with M2 polarization by mimicking pulmonary surfactant and simultaneously reduces TGF-β1 secretion (**Figure 5**) (153). This dual physico-chemical and biological regulatory mechanism highlights the multi-faceted efficacy of liposome therapy. Furthermore, surface modifications of liposomes (e.g., mannose ligands (154)) can specifically enhance macrophage uptake and modulate their polarization direction. When combined with the localized high-concentration advantage of inhalation administration (e.g., a 2 mg/kg nebulized dose outperforming a 60 mg/kg oral dose (153)), future developments may involve intelligent liposome platforms that integrate targeted delivery, polarization regulation, and combination therapy (e.g., siRNA-small molecule co-delivery strategies (155)). Such approaches could break through the current limitations of anti-fibrotic therapy from the perspective of multi-cellular interaction networks.

**Figure 6:**
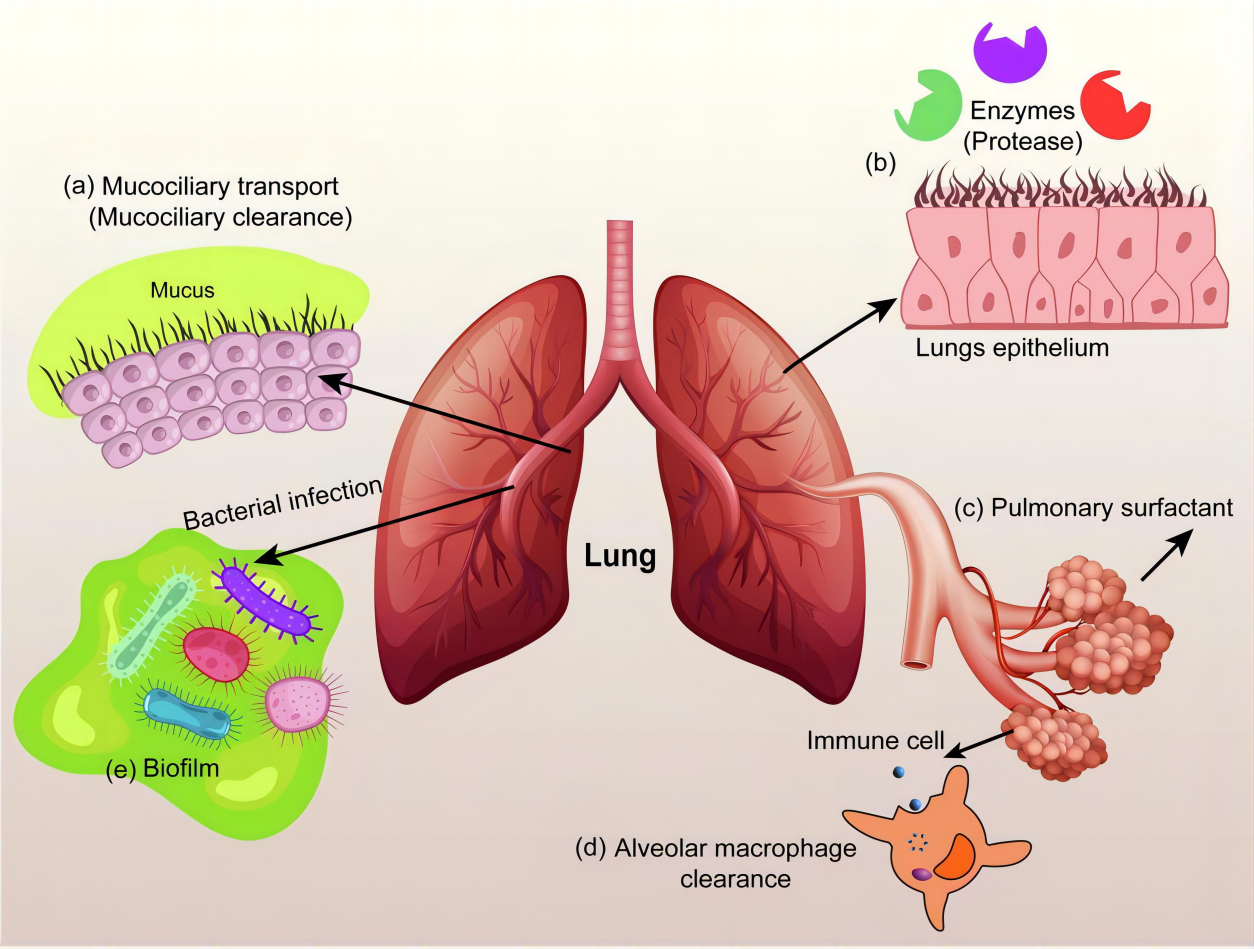
Inhalable antibiotic nanoformulations for the treatment of chronic respiratory diseases (160).

COPD not only severely impairs patients’ quality of life but also significantly increases the risk of cardiovascular events, recurrent respiratory failure, and susceptibility to lung cancer, thereby contributing to elevated overall morbidity and mortality (156). The pathological core of COPD involves macrophage polarization imbalance and chronic airway inflammation, yet conventional drug delivery systems struggle to precisely intervene in the immune microenvironment of affected areas. In response to this challenge, nanomedicine has emerged as a pioneering therapeutic strategy through precision drug delivery systems that molecularly target diseased tissues (157). It offers new avenues to enhance treatment efficacy while reducing reliance on conventional drugs and their associated adverse effects. Studies have shown that surface-modified (e.g., PEGylated) liposomes exhibit superior penetration capability and epithelial uptake efficiency in the pathological mucus of COPD, laying the foundation for targeting airway-resident macrophages (158). PEG modification not only reduces mucoadhesion through steric hindrance but may also influence macrophage phagocytic behavior by modulating liposomal surface properties. Specifically, PEGylated liposomes with a neutral charge and a nano-scale size (40–65 nm) are more readily internalized by macrophages, thereby enabling targeted delivery of anti-inflammatory drugs such as beclomethasone dipropionate or genetic regulators like miRNA/siRNA. This characteristic aligns well with the requirements for oligonucleotide delivery proposed by Li et al. (159): liposome-encapsulated silencing of M1 polarization-related genes (e.g., NF-κB or TNF-α) may reverse the hyperactivation of pro-inflammatory macrophages in COPD. Furthermore, antibiotic-loaded liposomes (e.g., tobramycin/colistin) developed by Zhang et al. (**Figure 6**) (160) not only target and eliminate pulmonary pathogens but may also break the “infection-inflammation” vicious cycle by modulating macrophage phagocytic function. Future directions may explore multifunctional designs, such as surface conjugation of CD206 antibodies to enhance M2 macrophage-specific targeting, combined with co-delivery of IL-10 mRNA and antibacterial agents to achieve dual “pro-repair/anti-infection” regulation. However, caution is warranted regarding the potential long-term impact on macrophage functional homeostasis. This could be mitigated by using biodegradable lipid materials to construct stimulus-responsive release systems that activate drug delivery exclusively within inflammatory microenvironments, thereby balancing efficacy and safety.

**Figure 7:**
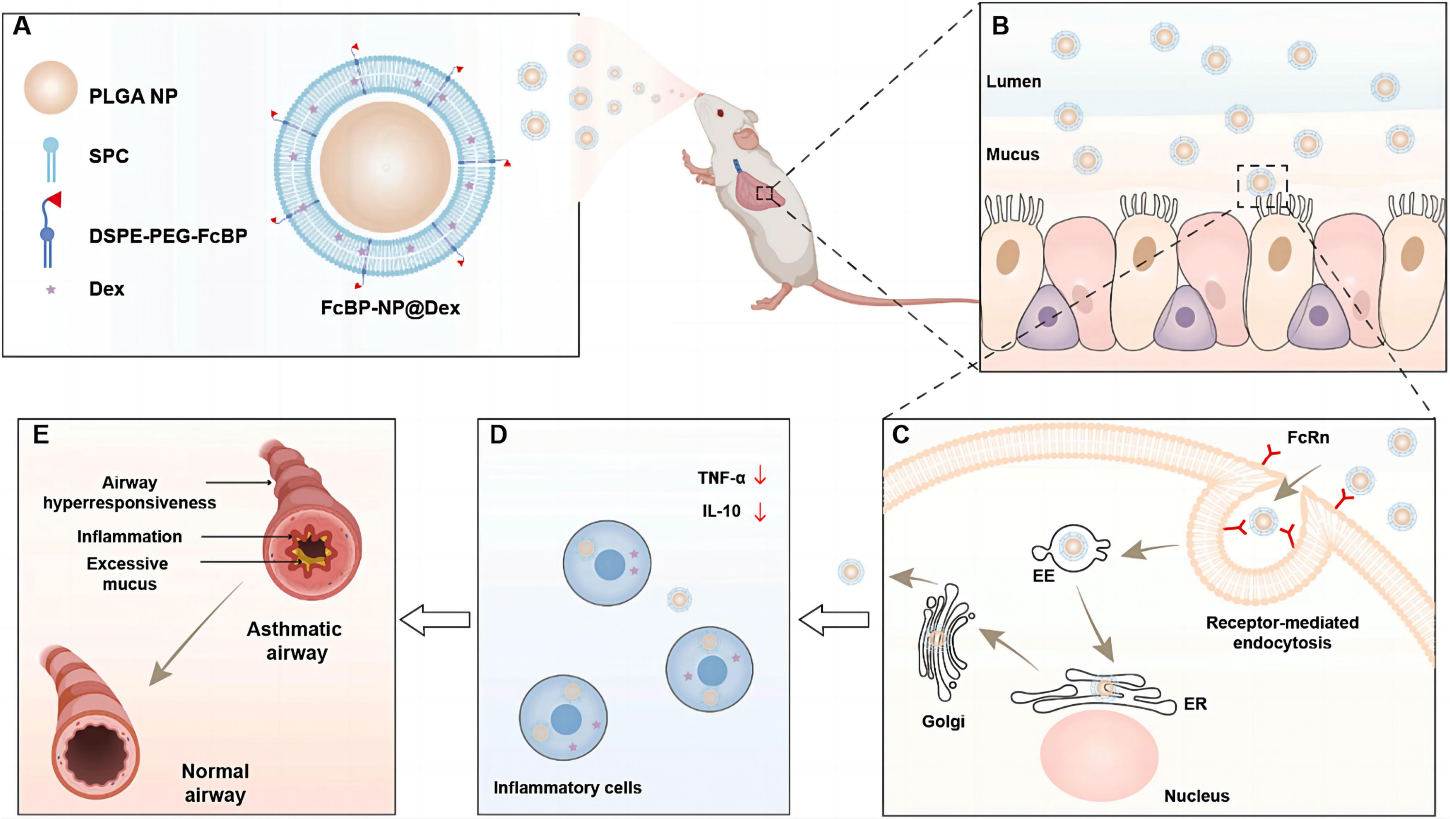
FcBP-functionalized PEG nanoparticles overcoming airway barriers and enhancing asthma therapy (167).

As central effector cells in asthma-related inflammatory regulation, macrophages contribute directly to airway hyperresponsiveness and amplification of inflammation through M2 polarization (161) or maturation defects (162). Over the past 15 years, significant advances in medical interventions have led to a substantial decline in asthma incidence and mortality, with most patients achieving adequate symptom control through conventional treatment regimens (163). However, current therapies remain insufficient for severe or refractory cases, where symptom management continues to pose major challenges. This unmet clinical need is driving the exploration of more precise and effective treatment strategies. Through multifunctional liposomal design, precise modulation of macrophage phenotypes has become achievable: MBD2 siRNA-loaded liposomes suppress the M2 polarization program in macrophages, thereby blocking the allergic inflammatory cascade at an upstream stage (161), while MPLA/Dex hybrid nanoparticles actively target macrophages via TLR4 ligands, simultaneously inhibiting pro-inflammatory phenotypes and promoting IL-10-mediated immune tolerance (164). Notably, intelligent modulation of liposome surface properties (165) can optimize pulmonary retention and transmembrane efficiency. For instance, highly hydrophilic liposomes prolong budesonide retention in alveolar macrophages, and cyclic peptide modifications targeting ICAM-1 (166) provide molecular guidance to enhance macrophage-specific uptake. Zhang et al. successfully prepared cyclopeptide-modified lipid nanoparticles (Pep-LNPs) that can precisely deliver siRNA to human and mouse epithelial cells, significantly reducing the expression of pro-inflammatory cytokines (TSLP), modulating asthma-related signaling pathways, decreasing MUC5AC mucin secretion, alleviating airway inflammation, lowering airway hyperreactivity, and improving asthma symptoms (166). Additionally, Yu et al. prepared PEG-coated PLGA-liposomes (PEG-NP) modified with FcBP to enhance targeting recognition capabilities (**Figure 7**) (167). Experiments showed that FcBP-NP@Dex efficiently delivered Dex to macrophages, exhibited significant anti-inflammatory effects, and demonstrated promising therapeutic outcomes in asthmatic mice. These synergistic innovations suggest that future systems may integrate “targeted delivery–phenotypic reprogramming–long-term regulation” into a unified liposomal platform. For example, co-delivery of MBD2 siRNA (161) and GM-CSF (162) could simultaneously rectify maturation defects and suppress aberrant polarization. Nonetheless, caution is warranted regarding potential long-term impacts on innate immune function, which may be mitigated through spatiotemporally controlled release technologies to balance therapeutic efficacy and immune homeostasis. In summary, although PF, COPD, and asthma exhibit distinct pathological features, they share a central link in chronic inflammation and dysregulation of the immune microenvironment, where macrophages play a critical role. In light of this, adopting multi-pronged strategies holds promise for fundamentally reversing the immune imbalance underlying the progression of these diseases. Although current research has only just unveiled the beginning of this field, it has already revealed the immense potential and fascinating prospects of nanomedicine-based approaches in tackling complex chronic respiratory diseases.

## 5 Diabetes mellitus

Metabolic disorders (MDs) represent a complex group of interconnected pathological conditions characterized by dysregulation in the metabolism of fundamental macronutrients including carbohydrates, lipids, and proteins (168). This disease spectrum encompasses a range of clinically significant conditions such as obesity, type 2 diabetes mellitus (T2DM), non-alcoholic fatty liver disease (NAFLD), hypertension, osteoporosis, chronic kidney disease, and cardiovascular disorders, all of which share common metabolic dysfunctions (169). The global impact of these conditions is profound, with diabetes mellitus alone affecting more than 415 million individuals worldwide, creating significant challenges for healthcare systems and socioeconomic structures (170). These disorders not only compromise patients’ quality of life through multiple organ system involvement but also contribute to substantial morbidity and mortality rates. The alarming prevalence of MDs highlights the urgent need for enhanced research efforts to develop more effective diagnostic, preventive, and therapeutic approaches. Given the extensive clinical implications and research significance, this section will particularly focus on diabetes mellitus (DM) as a representative metabolic disorder.

DM comprises a group of complex metabolic disorders characterized by chronic hyperglycemia resulting from either deficient insulin secretion, impaired insulin action, or both pathological mechanisms (171, 172). The growing global prevalence of diabetes presents a significant public health challenge, with millions affected by persistent elevated blood glucose levels (173, 174). These challenges have driven the urgent need for developing more effective therapeutic agents and improved drug delivery systems with enhanced precision and reduced adverse effects. In this context, liposome-based delivery systems have emerged as a promising approach, offering several potential advantages including versatile applicability, targeted delivery capabilities, and modifiable properties for optimized therapeutic outcomes. Liposomes have demonstrated significant potential in improving mitochondrial function and regulating blood glucose metabolism in diabetic mice. Wu et al. developed Nano-MitoPBN, a novel liposomal nanoparticle designed to enhance mitochondrial performance and promote hepatic oxidative metabolism (175). This formulation improves the efficiency of both glycolysis and the tricarboxylic acid cycle, thereby accelerating glucose metabolism and cellular uptake. In diabetic animal models, Nano-MitoPBN effectively reduces peripheral blood glucose levels and improves glucose tolerance, representing a promising therapeutic strategy for diabetes management.

**Figure 8:**
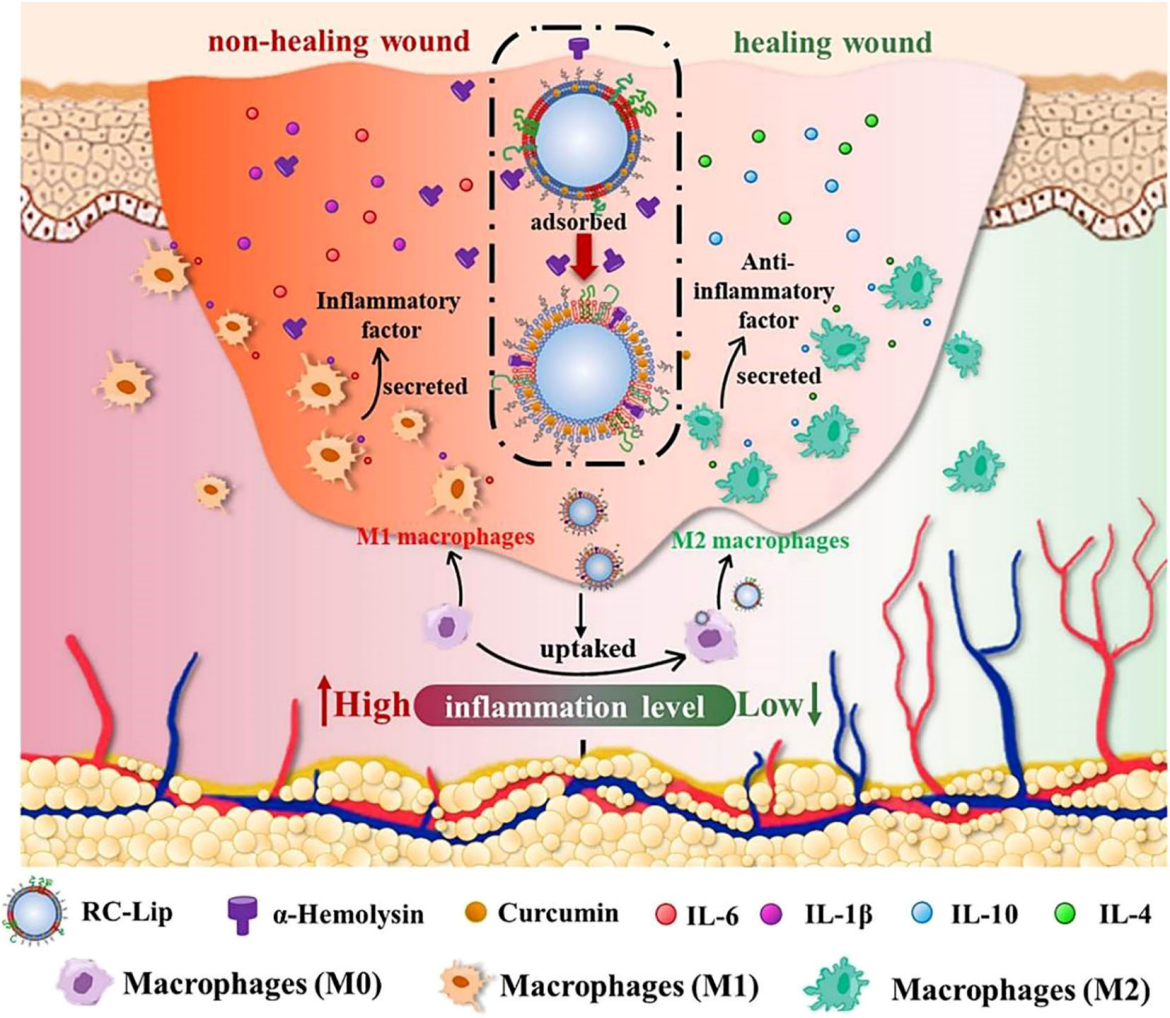
Mechanism of multimodal therapeutic hybrid liposomes in promoting wound healing in diabetes and infection (178).

Liposomes show promise in enhancing wound healing in diabetic patients. Diabetic wounds are particularly vulnerable to bacterial infection due to persistent hyperglycemia and elevated ROS levels, which significantly impair the healing process (176). These factors interact synergistically, worsening wound progression. Conventional therapeutic approaches can provide partial symptomatic relief through oral hypoglycemic agents for blood glucose control, intravenous antibiotics for infection management, and topical antiseptics for pathogen elimination. However, these interventions often prove insufficient to fully resolve the complexity of diabetic wounds (177). While these approaches provide temporary symptom management, they typically do not address the underlying mechanisms hindering wound repair. Excessive inflammation is a key obstacle in diabetic wound healing. To address this, Tang et al. designed red blood cell-mimicking liposomes (RC-Lips) loaded with curcumin, which neutralize bacterial toxin α-hemolysin, modulate M2 macrophage polarization, and fine-tune the inflammatory response, thereby accelerating diabetic wound healing (**Figure 8**) (178). Similarly, Liu et al. co-encapsulated a near-infrared-II (NIR-II) photothermal agent (IRC) and curcumin into thermosensitive liposomes, creating the Cur-IRC@PCM nanoplatform for precise and effective treatment of methicillin-resistant *staphylococcus aureus* (MRSA)-infected diabetic wounds (179). Furthermore, Wei et al. engineered Janus liposomes capable of reprogramming macrophage polarization and stimulating tissue regeneration. Using single-cell RNA sequencing and T-cell-deficient mouse models, they demonstrated the critical role of γδ T cells in M1/M2 macrophage switching (180). In summary, these liposome-based strategies represent a paradigm shift in diabetic wound management, moving from conventional symptomatic treatment to multi-mechanism-based synergistic intervention. Such platforms simultaneously address hyperglycemia, bacterial infection, oxidative stress, and immune dysregulation, demonstrating notable therapeutic superiority over traditional approaches. In the future, the efforts should focus on developing biomarker-responsive smart liposomes, optimizing combination therapies targeting multiple pathological pathways, and establishing standardized protocols for clinical evaluation of nanotherapeutics in diabetic wound healing.

## 6 Challenges and outlook

As key regulators of the innate immune system, macrophages play a dual role in the pathogenesis of chronic diseases, such as cardiovascular diseases, cancer, respiratory diseases, and diabetes. Macrophages can not only promote inflammation and tissue damage, but also participate in repair and homeostasis restoration through phenotypic polarization (e.g., transition from pro-inflammatory M1 to anti-inflammatory M2 phenotypes) (181, 182). Owing to the good biocompatibility (183), drug-loading capacity (184), and potential for targeted modification (185), liposomes have emerged as important tools for modulating macrophage functions (**Table 1**). However, the clinical translation of this strategy still faces multiple challenges, and future breakthroughs will require technological innovation and interdisciplinary collaboration.

**Table 1:** Macrophage-targeting drugs used in preclinical or clinical studies for chronic diseases.

Diseases	Lipsome	Size (nm)	Drug	Target	Effect	Refs.
Cardio-vascular	PLM-miR	/	miR-21	macrophage	Repair and reprogram inflammatory macrophages	(44)
	ST-MT@lipo2	207.4 ± .81	MLT (melatonin)	Monocytes and macrophages	Reduce the migration of circulating inflammatory monocytes	(45)
	PLP-RvD1	120.93 ± 2.99	RvD1	Monocyte	Enrich RvD1 and promote angiogenesis	(48)
	MTX-liposomes	111 ± 46	MTX	Inflamed tissue	Locally reduce the levels of inflammatory cytokines	(49)
	HA - ML@(H + R) NPs	/	Rosuvastatin, hydroxysaffron yellow A	CD44, macrophage cell membrane	Reverse the macrophages from M1 to M2 phenotype, down-regulate CD36	(66)
	HA - ML@ES NPs	216.3 ± 4	Shikonin (SKN), elomumab (Evol)	CD44	Inhibit endothelial cell glycolysis, reprogram the macrophage phenotype to restore cholesterol flow homeostasis	(67)
	Osteopontin modified nano-liposomes (CZALO)	/	L-arginine (L-Arg), cerium-zirconium oxide nanoparticles (CZ NPs)	Macrophage	Clear ROS and promote M2 polarization, generates NO	(68)
	EGCG/Cys/UDCA@VHPK - Lipo	191.2 ± 5.81	Epigallocatechin gallate (EGCG), cysteine (Cys), ursodeoxycholic acid (UDCA)	VCAM - 1	Promotes the dissolution and excretion of cholesterol, eliminates ROS, up-regulate ABCA1/ABCG1 to promote M2 polarization	(69)
	HMLRPP NPs	197.6 ± 5.12	Rosuvastatin (Pit), resveratrol (Res)	Macrophage cell membrane, CD44	Inhibit ferroptosis of macrophages, reduce lipid accumulation and inflammation	(70)
	DHA Liposomal	127 ± 7	Docosahexaenoic acid (DHA)	Macrophage	Phagocytosed by macrophages and exerts anti-inflammatory and antioxidant effects, promote M2 polarization	(77)
Cancer	TSPLs	~800	Recombinant Staphylococcal enterotoxin C2 (rSEC2) + paclitaxel	Lung targeting + tumor homologous targeting	Increase CD4^+^ and CD8^+^ T cells, induces apoptosis of tumor cells	(107)
	NPCD@ALN	/	Cisplatin + decitabine	ALN	Activate the caspase-3/GSDME pathway to induce pyroptosis of cells, trigger ICD	(108)
	si/F@RL	82.6	FTO protein inhibitor + CD47 siRNA	Macrophage	Inhibit the FTO protein and drive the repolarization of M2 to M1-type TAM	(114)
	ThioLipos	192	Thiopoton (TST, a FOXM1 inhibitor)	FOXM1/PD-L1 pathway	Inhibit the FOXM1 transcription factor and down-regulate PD-L1	(117)
	BLN	500-700	/	Macrophages/immune microenvironment	Reprogram M2-like macrophages to an M1-like phenotype	(118)
	Axi/siRNA@NGR-Lipo	156.2	Axitinib + PD-L1 siRNA	Tumor vascular endothelial cells/PD-L1	Targeting of tumor blood vessels, down-regulates PD-L1	(105)
	O3_PFD@Liposome	156.5	Ozone (O3)	Tumor cells/immune microenvironment	Generates reactive oxygen species (ROS), radiotherapy to induce ICD, and works in synergy with PD-1 blocking to inhibit tumors	(120)
	Oxaliplatin liposome	122	Oxaliplatin (ICD inducer) + ADU-S100 (STING agonist)	cGAS-STING pathway/immune microenvironment	Induced ICD, in synergy with STING agonists, converts M2 to M1 phenotype	(91)
	REV@SR780Fe@LEV-RS17	123.8 ± 2.8	SR780 (photosensitizer) + Fe³^+^ + RS17 peptide	Tumor microenvironment/cGAS-STING pathway	Activates cGAS-STING pathway	(128)
	RB-OL@M	126.4 ± 3.8	Imiquimod (R837) + Black phosphorus quantum dots (BPQDs)	Tumor site (macrophage homing)	Phototherapy, reprogramming TAM	(132)
	TMVL-I	160	Indocyanine green	PD-1/PD-L1 immune checkpoint	Blocks the PD-1/PD-L1 pathway, enhances the ICD effect	(136)
Respiratory	Liposomes loaded with SAB	100–200	Salvia acid B (SAB)	Lungs (inhalation delivery)	Inhibit inflammation and regulate the coagulation-fibrinolytic system	(150)
	Nin-lipo	198	Nintedanib	Alveolar macrophages	Polarize of M2 macrophages, inhibited TGF-β1	(153)
	Man-lipo	234.13 ± 1.76	Cryptotanshinone (CTS)	Macrophage	Inhibit NLRP3/TGF-β1 pathway and regulate MMP-9/TIMP-1 balance	(154)
	PEG-liposomes	40-65	Beclomethasone dipropionate (BDP)	Airway epithelium	Penetrate sputum and internalize into epithelial cells	(158)
	Tobramycin/colistin nanoformulations/amikacin liposomes	/	Tobramycin/colistin/amikacin	Drug-resistant pathogen	Enhance antibiotics and reduce their toxicity, targeting multi-drug resistant pathogens	(160)
	Mbd2 siRNA loaded liposomes	/	Mbd2 siRNA	Macrophage	Reduce the expression of Mbd2 and inhibit the polarization of M2 macrophages	(161)
	Pep-LNP	100-200	TSLP siRNA	ICAM-1 receptor at the top of airway epithelial cells	Downregulate TSLP, alleviate inflammatory cell infiltration, IL-4/IL-13 secretion and mucus production	(166)
	FcBP-PEG-NP	115-145	Dexamethasone	Neonatal Fc receptor	Maintain mucus penetrability and enhance intracellular internalization/transepithelial transport	(167)
Diabetes	Nano-MitoPBN	100 nm	/	Liver mitochondria	Reduces oxidative stress, boosts ATP synthesis, protects mitochondria	(175)
	RC-Lip	137.10 ± 1.43	Curcumin	α-hemolysin	Downregulates IL-1β, upregulates IL-10	(178)
	Cur-IRC@PCM	110	Curcumin	MRSA	Photothermal therapy and Antibacterial activity	(179)

The primary technical bottleneck lies in the limitations of liposomal targeted delivery and stability. First, insufficient targeting precision is a key constraint. Although surface modifications can enhance directional delivery capabilities (186), liposomes still struggle to efficiently recognize and specifically accumulate in target macrophages within highly heterogeneous *in vivo* environments (187), compromising treatment accuracy. Second, liposomes are susceptible to adsorption by plasma proteins, enzymatic degradation, and interference from blood components during systemic circulation, leading to structural integrity loss and premature drug leakage (188). This not only reduces reprogramming efficiency but may also increase off-target toxicity risks due to non-specific release.

Although surface modifications (e.g., antibodies, peptides) can achieve macrophage-targeted delivery, the circulation time of liposome systems is significantly compromised by rapid clearance via the reticuloendothelial system (RES), resulting in predominant accumulation in the liver/spleen and insufficient deposition at disease sites (189). Therefore, there is an urgent need to improve targeting precision. By the way, high shear stress in atherosclerotic plaques hinders the stable retention of liposomes, while in myocardial infarction models, rapid endothelial barrier repair before the peak of macrophage infiltration leads to low systemic delivery efficiency (44). Furthermore, as carriers for RNA therapies (e.g., miRNA), liposomes require substantial improvements in loading and release efficiency, facing challenges such as degradation by serum RNases and insufficient endosomal escape, which hinder cytoplasmic delivery (190). Stimuli-responsive liposomes (e.g., pH- or enzyme-sensitive types) exhibit poor spatiotemporal control in complex pathological microenvironments, often resulting in burst release or abnormal drug retention (191).

In terms of immunogenicity, although liposomes generally exhibit good biocompatibility (192), certain surface modifications or encapsulated drugs may enhance their immunogenicity. This not only facilitates rapid clearance by the immune system, reducing therapeutic efficacy, but may also trigger adverse reactions such as allergies, posing risks to patient safety. For instance, cationic liposomes, while enhancing cellular uptake, may activate the complement system and induce complement activation-related pseudoallergy (CARPA), characterized by histamine release and acute inflammation (193), presenting immunogenicity and toxicity concerns. Systemic immune activation may lead to severe immune-related adverse events, such as cytokine release syndrome (CRS), neurotoxicity, or autoimmune tissue damage (194, 195). Furthermore, a more comprehensive evaluation of the long-term safety and immunogenicity of liposome components and their metabolites is required. Additionally, excessive uptake of liposomes by macrophages may inhibit phagocytic function, impair host antimicrobial defense, and result in immunosuppression risks. Regarding long-term efficacy, data on the application of this strategy for chronic disease treatment remain limited (196). It is unclear whether the reprogrammed state can be sustained long-term or what the enduring impact on disease progression might be. Prolonged use may also lead to macrophage dysfunction and potential side effects, significantly limiting its clinical translation prospects. Moreover, there is a lack of long-term toxicity data on liposome components and their metabolites, particularly a deficiency in lifetime longitudinal safety studies.

There are also limitations in therapeutic mechanisms and disease models, since macrophage polarization regulation exhibits duality. For example, DHA- or miR-21-loaded liposomes induce M2 polarization to alleviate inflammation, but excessive suppression of the M1 phenotype in advanced plaques may impair pathogen clearance capacity and increase the risk of plaque rupture (197). Different lipid components yield significantly divergent therapeutic effects: anionic liposomes promote cholesterol efflux from foam cells (189) whereas cationic liposomes instead enhance inflammatory cytokine secretion (198). There are complexities in therapeutic strategies and clinical applications. Due to inter-patient heterogeneity in tumors (199), significant differences exist in the phenotype and distribution of TAMs among different patients (200), across various tumor types, and even within the same tumor. Universal targeting strategies may therefore fail to effectively cover all relevant immunosuppressive macrophage subsets. Furthermore, current regulatory strategies remain relatively simplistic, leading to limited efficacy or phenotypic reversal. Even if TAMs are successfully “reversed” from the M2 to the M1 phenotype via liposomes, the highly immunosuppressive tumor microenvironment may cause them to revert to a pro-tumor phenotype, resulting in transient and unsustainable therapeutic effects (201, 202). Additionally, single-target therapeutic strategies face limitations in efficacy. Most approaches focus on a single signaling pathway, but tumor immunosuppression results from complex interactions within multiple signaling networks. Blocking one pathway can easily be bypassed by compensatory mechanisms, leading to limited efficacy or drug resistance. Moreover, the complexity of combining these strategies with existing clinical treatments further complicates translation. While combination with chemotherapy, radiotherapy, or immune checkpoint inhibitors is most likely, this makes clinical trial design extremely complex (203) particularly in determining the optimal dosing timing and sequence, while also increasing the risk of unpredictable synergistic toxicities. A critical translational gap exists between disease models and human pathophysiology: the immune microenvironment in mouse atherosclerosis models (e.g., ApoE^-/-^) differs significantly from that of human plaques (204), particularly in terms of macrophage subtype complexity. This explains why anti-inflammatory strategies successful in animal models frequently fail in clinical trials. In myocardial ischemia-reperfusion injury models, due to differences in cardiovascular anatomy, the targeting efficiency of liposomes in large animals is markedly lower than in rodents, limiting the predictive value of preclinical data.

The core challenges in the clinical translation of liposomes lie in the barriers associated with production, preparation, and quality control. The manufacturing process for complex liposomes (e.g., those modified with antibodies, peptides, or exosomes) is highly intricate, making it difficult to precisely control particle size, encapsulation efficiency, and batch-to-batch consistency, which severely restricts their industrial-scale production and clinical applicability. Additionally, technologies such as directional membrane protein integration pose challenges for GMP compliance, and large-scale production entails high costs, limiting scalability. Regulatory frameworks lag behind technological advancements, and existing drug classification systems struggle to clearly define multi-component or surface-engineered liposome products, creating bottlenecks in the approval of combination therapies. Traditional RECIST criteria may fail to accurately capture delayed immune responses or changes in the immune status of the TME (205), leading to misinterpretation of early clinical trial results. Furthermore, the biological behavior of liposomal drugs is complex. The timing, location, and mechanisms of drug release remain poorly understood, and current analytical methods are inadequate for distinguishing between released drugs and those still encapsulated within the carrier, hindering precise efficacy evaluation. The production costs of targeted liposome therapies are significantly higher than those of conventional drugs, and when combined with the long-term treatment requirements for cardiovascular diseases, this imposes a substantial economic burden on healthcare payment systems.

Despite numerous challenges, liposome-mediated macrophage reprogramming holds broad clinical translation potential through multidimensional strategy optimization and interdisciplinary collaboration. First, it is essential to strengthen collaboration between pharmaceutical researchers and clinicians to identify ideal candidate drugs suitable for liposomal formulation development that address clinical needs. Second, basic research should focus on elucidating the physicochemical and biological principles underlying liposome preparation and therapeutic mechanisms. A deeper understanding of drug-lipid interactions, molecular dynamics during liposome self-assembly, and liposome-biofluid-cell interactions will facilitate the design of more efficient and safer liposomal delivery systems. Clarifying the pharmacokinetic behavior of liposomal drugs will provide critical guidance for optimizing therapeutic strategies.

In terms of design, smart targeted liposomes can be developed by utilizing targeting ligands such as aptamers (206), antibodies (207) or peptides (208) to specifically recognize macrophage surface markers, thereby enhancing targeting efficiency. Additionally, designing liposomes that respond to the disease microenvironment enables precise drug release (209). In terms of preparation processes, microfluidic technology (210, 211) shows increasingly broad prospects in liposome production. The introduction of techniques such as microfluidics (212) facilitates precise control over particle size and morphology, enabling stable and continuous large-scale manufacturing while ensuring batch consistency through real-time quality monitoring. These advanced manufacturing methods can significantly improve production efficiency and reduce costs, but rigorous quality control protocols must be established to ensure efficacy and safety standards.

In terms of therapeutic mechanism research and personalized treatment, The integration of multi-omics technologies such as transcriptomics (213), proteomics, and metabolomics (214), enables systematic elucidation of key signaling pathways and targets during the reprogramming process,. Single-cell analysis techniques (215) should be applied to uncover macrophage subset heterogeneity and differences in liposome intervention effects, providing a basis for personalized treatment. Regarding safety and efficacy, immune modulation strategies need to be developed, such as optimizing liposome surface modifications (216) to reduce immunogenicity or combining with immunomodulators to enhance therapeutic outcomes. Concurrently, long-term clinical follow-up studies should be conducted to systematically evaluate efficacy and safety, while leveraging clinical big data and artificial intelligence to optimize treatment regimens.

Finally, interdisciplinary collaboration should be strengthened by integrating expertise from biomedical science, materials science, chemical engineering, and other fields to drive technological innovation. Through deep integration of industry, academia, and research, the clinical translation and application of liposome technology in macrophage reprogramming therapy can be accelerated. Enhanced collaboration among pharmacology, clinical medicine, materials science, and regulatory science will facilitate the selection of ideal candidate drugs, optimization of treatment strategies, and advancement of regulatory frameworks. Establishing long-term follow-up study systems, combined with clinical big data and artificial intelligence, will enable systematic evaluation of efficacy and safety, ultimately achieving widespread application of liposome-mediated macrophage regulation therapy in the treatment of chronic diseases.

Looking ahead, overcoming these challenges requires a multifaceted approach. Strengthening collaboration between pharmaceutical researchers and clinical physicians is crucial. While pharmaceutical researchers focus on developing novel liposome formulations, clinicians possess deeper insights into patients’ actual needs and treatment responses. By working together, they can identify the optimal candidate drugs that meet clinical demands, ensuring liposome therapies are better aligned with real-world treatment scenarios.
